# Race and Cognitive Health Among Older Americans: The Role of Debt and Asset Profiles During Preretirement Age

**DOI:** 10.1093/geroni/igaf122.1412

**Published:** 2025-12-31

**Authors:** Chioun Lee, Dana Glei, Soojin Park

**Affiliations:** University of California, Riverside, Riverside, California, United States; Georgetown University, Washington, District of Columbia, United States; University of California, Riverside, Riverside, California, United States

## Abstract

**Objectives:**

Low-cost debt can potentially enhance wealth and indirectly benefit health, yet Black Americans disproportionately lack this type of debt, which may constrain their ability to accumulate wealth throughout their lives and across generations. Our objectives are to develop a novel debt–asset measure, use it to quantify the Black–White differential in debt–asset profiles, and estimate its contribution to the racial gap in cognition.

**Methods:**

Using the Health and Retirement Study (1998–2020), we grouped individuals based on debt and asset information during the preretirement period of ages 55 to 61, including the absence of debt and the relative amount of debt compared to assets. Linear mixed models were used to examine the extent to which cognition in later life (ages 62–80) differs across these debt–asset profiles and its role in explaining the racial disparity in cognition.

**Results:**

Compared with Whites, Blacks were more likely to fall into categories characterized by high debt-to-asset ratio (DAR) or limited asset ownership. Low-asset non-borrowers displayed the poorest cognition, followed closely by high-DAR borrowers. The Black–White differential in debt–asset profiles contributed to the racial gap in cognition.

**Discussion:**

There were two unfavorable debt–asset profiles: high debt relative to assets and little or no debt due to a lack of assets, which was more prevalent among Blacks than Whites. We discuss how institutional and structural racism shapes Black–White disparities in debt–asset profiles, such as limited access to borrowing opportunities, thereby contributing to health inequalities, including cognition.

